# Protocol for a randomised control trial of methylnaltrexone for the treatment of opioid-induced constipation and gastrointestinal stasis in intensive care patients (MOTION)

**DOI:** 10.1136/bmjopen-2016-011750

**Published:** 2016-07-13

**Authors:** Parind B Patel, Stephen J Brett, David O'Callaghan, Aisha Anjum, Mary Cross, Jane Warwick, Anthony C Gordon

**Affiliations:** 1 Centre for Perioperative Medicine and Critical Care Research, Imperial College Healthcare NHS Trust, London, UK; 2 Section of Anaesthetics, Pain Medicine and Intensive Care, Department of Surgery and Cancer, Imperial College London, London, UK; 3 Imperial Clinical Trials Unit, Faculty of Medicine, School of Public Health, Imperial College London, London, UK; 4 Warwick Clinical Trials Unit, University of Warwick, Coventry, UK

## Abstract

**Introduction:**

Gastrointestinal dysmotility and constipation are common problems in intensive care patients. The majority of critical care patients are sedated with opioids to facilitate tolerance of endotracheal tubes and mechanical ventilation, which inhibit gastrointestinal motility and lead to adverse outcomes. Methylnaltrexone is a peripheral opioid antagonist that does not cross the blood–brain barrier and can reverse the peripheral side effects of opioids without affecting the desired central properties. This trial will investigate whether methylnaltrexone can reverse opioid-induced constipation and gastrointestinal dysmotility.

**Methods:**

This is a single-centre, multisite, double-blind, randomised, placebo-controlled trial. 84 patients will be recruited from 4 intensive care units (ICUs) within Imperial College Healthcare NHS Trust. Patients will receive intravenous methylnaltrexone or placebo on a daily basis if they are receiving opioid infusion to facilitate mechanical ventilation and have not opened their bowels for 48 hours. All patients will receive standard laxatives as per the clinical ICU bowel protocol prior to randomisation. The primary outcome of the trial will be time to significant rescue-free laxation following randomisation. Secondary outcomes will include tolerance of enteral feed, gastric residual volumes, incidence of pneumonia, blood stream and *Clostridium difficile* infection, and any reversal of central opioid effects.

**Ethics and dissemination:**

The trial protocol, the patient/legal representative information sheets and consent forms have been reviewed and approved by the Harrow Research Ethics Committee (REC Reference 14/LO/2004). An independent Trial Steering Committee and Data Monitoring Committee are in place, with patient representation. On completion, the trial results will be published in peer-reviewed journals and presented at national and international scientific meetings.

**Trial registration number:**

2014-004687-37; Pre-results.

**Strengths and limitations of this study**

- Double-blind, placebo-controlled, randomised trial.
- Testing methylnaltrexone to treat an important patient-focused outcome (constipation and gastrointestinal stasis) in critical care.
- Limited sample size to answer other clinical outcomes.

## Introduction

### Background and rationale

Bowel dysfunction in the intensive care unit (ICU) represents an important problem in critical care, with up to 70% of patients suffering from constipation.^1^ There is increasing evidence that opioids contribute to perioperative and ICU bowel dysfunction.^2^ Other studies demonstrate that bowel dysfunction in the critically ill is associated with adverse outcomes including delay in gastric emptying leading to increased gastro-oesophageal reflux and aspiration, decreased enteral feeding, delayed ICU discharge and increased mortality.^3–5^ While bowel dysfunction in critically ill patients is multifactorial and some component is due to the general effects of complex critical illness, exogenous and endogenous opioids contribute to this bowel dysmotility.^6^ Restoration of normal gastrointestinal (GI) function is essential for establishing enteral feeding; it also protects against the bacterial translocation, alleviates GI discomfort due to constipation and shortens ICU stay.^7^

Potential therapeutic inroads have been made in addressing this problem. Naloxone, a competitive opioid antagonist, is most commonly administered systemically to counteract the central and peripheral effects of opioids. When administered enterally in high doses, naloxone has been found to have benefit in the critical care setting, with improved gastric emptying and reduced ventilator-associated pneumonia rates.^8^ Unfortunately in clinical practice, the use of naloxone is limited with large doses required when administered enterally, and the fact that a large proportion of those with gastric stasis are unable to tolerate the nasogastric naloxone itself. Of course, administering the drug via any other route would antagonise the desired central therapeutic effects (analgesia and sedation) in critical care patients.

Methylnaltrexone is a recently approved peripheral μ-opioid receptor antagonist. It is a quaternary ammonium compound with a positive charge, which limits its ability to cross the blood–brain barrier. Unlike tertiary opioid antagonists such as naloxone or naltrexone, methylnaltrexone does not reverse centrally mediated analgesia or precipitate withdrawal. It is commercially available in prefilled syringes as a sterile, clear and colourless to pale yellow aqueous solution (Salix Pharmaceuticals, Raleigh, North Carolina, USA). The chemical name for methylnaltrexone bromide is (*R*)-*N*-(cyclopropylmethyl) noroxymorphone methobromide. The molecular formula is C_21_H_26_NO_4_Br, and the molecular weight is 436.36.

The efficacy and safety of methylnaltrexone in the treatment of opioid-induced constipation (OIC) have been evaluated in two multicentre, randomised, double-blind, placebo-controlled phase III trials involving adults with advanced illness (life expectancy of 1–6 months) who were receiving palliative care.^9^ ^10^ The majority of patients had incurable cancer, but other diagnoses included cardiovascular disease, chronic obstructive pulmonary disease or emphysema and Alzheimer's disease or dementia.

Trial inclusion criteria included patients taking stable doses of opioids and laxatives for ≥3 days and subsequent OIC. Throughout all study periods, patients maintained their usual laxative regimen. The primary end points were rescue-free laxation, defined as a bowel movement within 4 hours of the first dose of methylnaltrexone. Secondary end points included time to laxation, pain scores, opioid withdrawal symptoms and adverse events.

The landmark published trial,^9^ compared methylnaltrexone 0.15 mg/kg (n=62) with placebo (n=71), administered on alternate days for 2 weeks. In the second week, the dose was increased to 0.3 mg/kg if the patient had fewer than three bowel openings by day 8. Methylnaltrexone improved the laxation rate within 4 hours of the first dose compared with placebo (48% vs 15% (p <0.001)). Of the patients who did respond within 4 hours of the first dose, half responded within 30 min. The study also showed that 52% of all patients taking methylnaltrexone had rescue-free laxation within 4 hours, when compared with 8% in the placebo group (p<0.001).

The efficacy of methylnaltrexone in the palliative care setting has been further confirmed, with a study that compared single subcutaneous doses of methylnaltrexone 0.15 mg/kg (n=47) or 0.30 mg/kg (n=55) with placebo (n=52).^10^ Methylnaltrexone significantly improved the laxation rate within 4 hours of dosing (62% for 0.15 mg/kg and 58% for 0.30 mg/kg vs 14% for placebo (p<0.0001 for each dose vs placebo)). The median time to laxation was shorter in the group administered methylnaltrexone (70 and 45 min for the 0.15 and 0.30 mg/kg groups, respectively, compared with placebo (>24 hours) (p<0.0001 for each dose vs placebo)).

While methylnaltrexone is approved for the treatment of OIC in advanced illness in palliative care patients, its use in the medical ICU has been limited and largely anecdotal. Case reports have reported an immediate effect of methylnaltrexone administration on bowel motility. In one report, methylnaltrexone was given intravenously to a critically ill patient with significant burns.^11^ The purpose of that use was to facilitate feeding, although bowel motility was also restored. After 4 days of no appreciable bowel function, there was instantaneous improvement in bowel sounds, flatus, gastric residuals and subsequently feeding. In another case, a patient with a palliative stoma and a long history of heroin abuse demonstrated no bowel function and significant distension 7 days after stoma formation.^12^ Within 15 min of methylnaltrexone (subcutaneous injection), there was a brisk output of over 1 L from the stoma. Both of these patients were receiving high doses of opioids. Additionally, a recent case report in a critically ill neonate with complex congenital heart disease complicated by 8 days of bowel dysmotility following ileosigmoid anastomosis demonstrated that methylnaltrexone (0.15 mg/kg subcutaneously) restored bowel function within 15 min of injection.^13^ The child was receiving a fentanyl infusion of 2 µg/kg/hour. A further case series was presented as an abstract, with patients from burns, cardiac and surgical ICUs being successfully treated with methylnaltrexone subcutaneous injections.^14^ These cases suggest that methylnaltrexone may significantly alleviate bowel dysfunction associated with the use of high doses of opioids in ICU patients.

In addition, we carried out a retrospective chart review of 88 non-surgical critical care patients receiving fentanyl infusions at the Hammersmith Hospital, Imperial College Healthcare NHS Trust over a 10-week period (1 September—15 November 2009).^15^ Fifteen patients met the criteria of failure to laxate within 72 hours despite treatment with senna and sodium docusate. Eight of these patients subsequently received conventional rescue therapy (combination of sodium picosulfate (5 mg) and two glycerin suppositories (4 g)), while seven patients received methylnaltrexone (subcutaneous injection, 0.15 mg/kg). Six of seven methylnaltrexone patients responded to one or two doses with laxation within 24 hours versus 0/8 for conventional rescue therapy (p=0.001). All methylnaltrexone patients, but only 4/8 of patients administered conventional rescue therapy, progressed to full target enteral feeding (p=0.076) within 24 hours. ICU mortality was 2/7 for methylnaltrexone vs 4/8 for standard therapy (p=0.61). There were no adverse effects from either rescue laxative therapies. These encouraging results further support the use of methylnaltrexone in critical care patients.

The use of opioids can also have an impact on infection. Exogenous opioids are known to have inhibitory effects on immune responses including T-lymphocyte function,^16^ B-lymphocyte function,^17^ natural killer cell activity^18^ as well as mononuclear cell proliferation, differentiation^19^ and phagocytosis.^20^

Thus, opioids may modulate the immune response through interaction with their receptors. As well as being present centrally, these receptors have been identified in peripheral nerves, and their endogenous peptide ligand is expressed on granulocytes, macrophages and lymphocytes.^21^ While yet to be established, the general effect of opioids is thought to be immunosuppressive.^22^

Infection is a major problem in critically ill patients with up to 37.4% of patients demonstrating sepsis in ICU. Common organisms include *Staphylococcus aureus* (30%, including 14% methicillin resistance), Pseudomonas spp (14%) and *Escherichia coli* (13%). Pseudomonas spp have been shown to be independently associated with increased mortality rates.^23^ Patients with sepsis have more severe organ dysfunction, longer ICU and hospital lengths of stay, and higher mortality rate than patients without sepsis. In animal studies, direct exposure of *Pseudomonas aeruginosa* to morphine in vitro showed that morphine transforms the bacteria to a more virulent phenotype that is attenuated in part by methylnaltrexone.^24^ If the peripheral effects of opioids are reversed in critical care patients, there could be an even more dramatic improvement in infection and patient outcome compared to simply reversing the GI side effects.

There are considerable safety data available on the use of methylnaltrexone. In phase III trials,^9^ ^10^ subcutaneous methylnaltrexone was well tolerated in patents with OIC and an advanced illness. The most common adverse effects reported for all doses of methylnaltrexone are abdominal pain, nausea, diarrhoea, flatulence, dizziness, injection site reactions and hyperhidrosis. None of the reported serious adverse events (SAEs) were attributed to the study drug.

Rare cases of GI perforation have been reported in patients with advanced illness and conditions that may be associated with localised or diffuse reduction of structural integrity in the wall of the GI tract (ie, cancer, peptic ulcer and Ogilvie's syndrome). Perforations have involved varying regions of the GI tract, for example, stomach, duodenum and colon.^25^ The Food and Drug Administration (FDA) recommends that methylnaltrexone is used with caution in patients with known or suspected lesions of the GI tract and is contraindicated in bowel obstruction and acute abdominal illness. Therapy should be discontinued if patients develop severe, persistent and/or worsening abdominal symptoms.^26^

There was no evidence of systemic opioid withdrawal or significant changes in pain scores throughout the phase III studies in palliative care or the retrospective pilot study in critical care.^15^

Methylnaltrexone is licensed for subcutaneous administration in palliative care patients as these groups of patients do not routinely have intravenous access and it can be self-administered subcutaneously. Many trials and case reports have demonstrated that intravenous administration is safe and efficacious.^11^ ^27^ ^28^ The pharmacokinetics of intravenous administration are well understood and predictable. In healthy volunteers, repeated administration of intravenous methylnaltrexone is well tolerated, with no significant adverse events or changes in opioid subjective ratings and no clinically noteworthy alterations in pharmacokinetics.^29^ In the ICU, all patients have intravenous catheter in place with 1:1 nursing, and furthermore, many are oedematous due to their underlying critical illness, justifying the use of the intravenous route as more appropriate.

Therefore, the rationale for the current study is that constipation and gut dysfunction are a major concern in intensive care patients. Reversal of this would lead to patient benefit.^30^ Methylnaltrexone has been shown to be beneficial in treating OIC in patients with advanced illness who are receiving palliative care when response to laxatives has not been sufficient.^9^ We hope to replicate the beneficial effects of methylnaltrexone in ICU patients. There may also be additional benefits in reducing infection and immunosuppression, and hence an overall improvement in patient outcome.

### Objectives

The primary objective of the study is to assess the efficacy of methylnaltrexone in inducing laxation in ICU patients sedated with opioid infusions.

The secondary objectives include observing whether the use of methylnaltrexone leads to increased opioid requirements through central nervous system penetration and antagonism, and assessing whether there are additional benefits such as reduced gastric stasis, improved enteral feeding and a reduction in infection; and finally to assess the safety and side effect profile of intravenous methylnaltrexone in ICU patients.

Plasma and serum will also be stored and further analysed for cytokine levels, metabolic profiles and leucocyte function assays performed to further investigate the mechanism of the immune effects of opiates and subsequent reversal.

### Trial design

**Figure 1:**
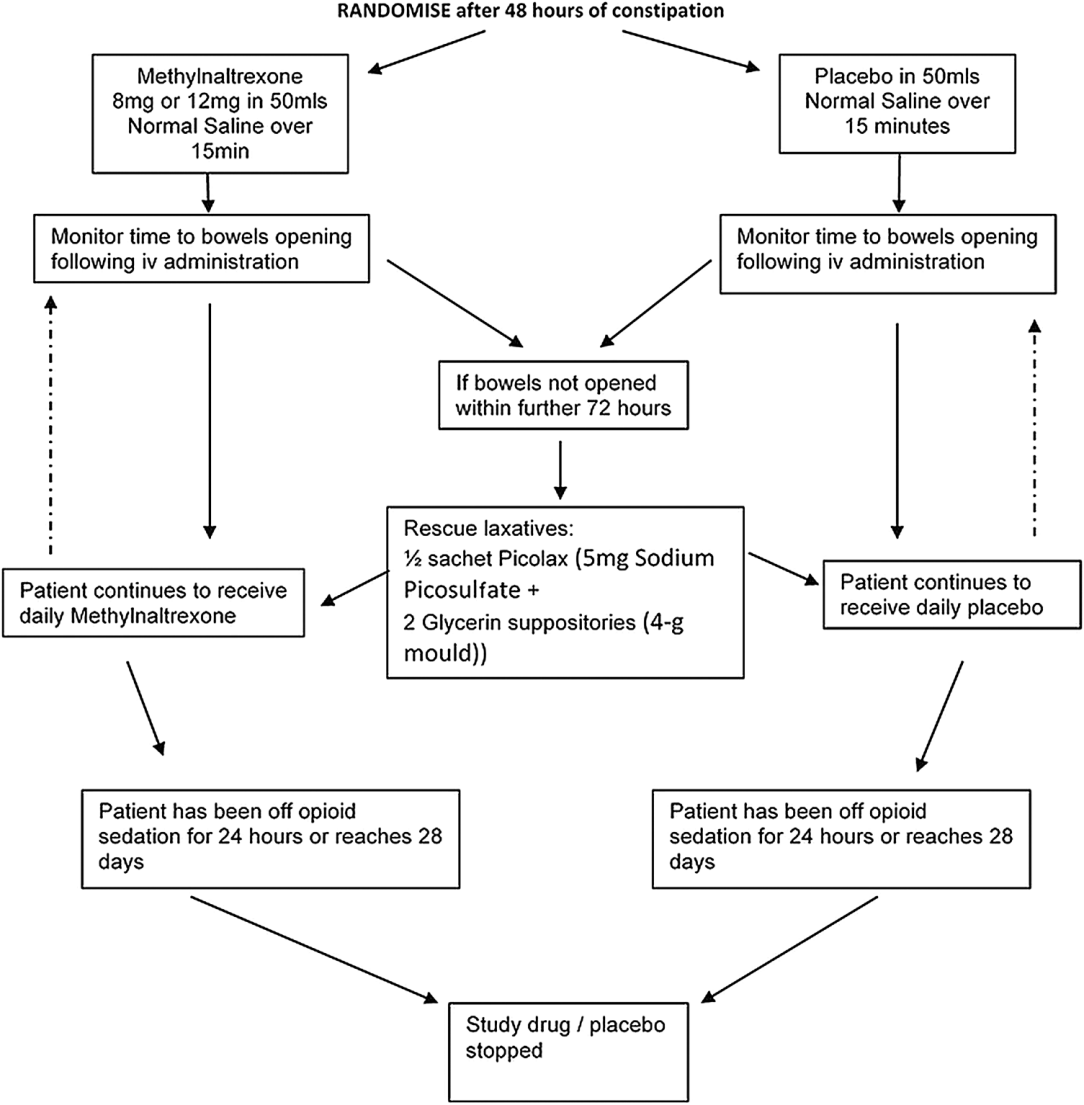
Flow chart. Adult critically ill patients sedated with and expected to remain on opioids for a further 24 hours, who have not opened their bowels for 48 hours. All patients are receiving standard ICU bowel care.

The study is an interventional, double-blind, randomised, placebo-controlled trial (see figure 1).

## Methods: Participants, interventions and outcomes

### Study setting

The study will be conducted in the ICUs within Imperial College Healthcare NHS Trust. The three hospitals are tertiary academic centres: Hammersmith Hospital, Charing Cross Hospital and St. Mary's Hospital. Further ICUs across other NHS Trusts may be considered at a later date.

### Eligibility criteria

All patients who are clinically constipated and receiving an opioid infusion will be screened against the inclusion and exclusion criteria for eligibility of the study.

The inclusion criteria are as follows:

- Men and women aged ≥18 years.
- Following ICU admission, sedated with opioids and requiring invasive ventilator support.
- Scheduled for continuous infusion/administration of opioid analgesics for at least a further 24 hours.
- Constipated (not opened bowels for a minimum 48 hours).
- Access for enteral administration of medications and gastric tube feeds.
- Initiation of gastric tube feeds.
- Patient weight of 38–114 kg (this allows pre preparation of drug with either 8 or 12 mg).

The exclusion criteria are as follows:

- Known to be pregnant.
- Patients with end-stage renal failure requiring dialysis prior to admission.
- Diarrhoea on admission.
- GI tract surgery within 8 weeks prior to ICU admission.
- Presence of ileostomy or colostomy.
- Mechanical GI obstruction.
- Suspected acute surgical abdomen.
- History of Crohn's disease or ulcerative colitis.
- Receiving palliative care or not expected to survive >12 hours.
- Severe chronic hepatic impairment (Child Pugh Class C).
- Suspected hepatic encephalopathy.
- Known to have received another investigational medicinal product within 30 days or currently in another interventional trial that might interact with the study drug or previously enrolled into MOTION.
- Known hypersensitivity to the study drug or any of its excipients.

### Interventions

All patients will be sedated to facilitate mechanical ventilation. The standard sedative regimens of the ICU will be followed, titrated by the bedside nurse and clinical team to the patient's need and the RASS (Richmond Agitation Sedation Score). The standard sedation will include an opioid (remifentanyl, fentanyl or morphine) and a hypnotic agent (propofol or midazolam).

All patients will be receiving standard ICU bowel care prior to study enrolment as part of the departmental bowel care policy.

Patients will be randomised to either treatment group or control group. The patient will remain in this group for the duration of the study.

Treatment group

- As per the Summary of Product Characteristics, patients weighing 38–61 kg will receive 8 mg (0.4 mL) methylnaltrexone diluted in 50 mL of 0.9% saline.
- Patients weighing 62–114 kg will receive 12 mg (0.6 mL) methylnaltrexone diluted in 50 mL of 0.9% saline.
- Treatment will be administered over 15 min via an indwelling intravenous catheter. The dose will be based on estimated actual body weight.

Control group

- Placebo (saline) prepared in identical syringes to study drug containing 50.4 or 50.6 mL of 0.9% saline.
- Placebo will be administered over 15 min via an indwelling intravenous catheter.

All patients

- The study drugs will be supplied to the ICU by pharmacy as specific research study drugs and they will be stored in separate research cupboards at room temperature. The study drug will be drawn up, labelled and administered by the research nurse on duty at that site. He/she will be non-blinded for the remainder of the study. He/she will not be involved in monitoring or collecting clinical outcome data.
- The study outcome measures are routinely collected and recorded by the bedside nurses and medical team, who will remain blinded to treatment allocation for the duration of the study. The study drug (active drug or placebo) will be prescribed on the patient drug chart by the clinical staff as per each ICU's policy, with blinding maintained.
- The patient will continue to receive the study drug at the same time on a daily basis, until the patient has been free of opioids for 24 hours or at 28 days.

Rescue therapy

- If a patient allocated to either arm fails to open their bowels within 72 hours of receiving study infusion, then rescue laxatives of a combination of sodium picosulfate (5 mg) and two glycerin suppositories (4 g) will be administered. The patient will continue to receive the study drug.

Other therapy

- If patients have high gastric aspirates and are not deemed to be absorbing enteral feed, then they will be administered prokinetics (erythromycin 250 mg intravenous four times a day and metoclopramide 10 mg intravenous three times a day) as per standard ICU protocol. These will be prescribed by the treating clinicians (blinded to study drug).
- All patients will receive the standard hospital approved enteral feed administered to a target infusion rate calculated by the treating ICU dietician.

Withholding study drug

- If the patient develops diarrhoea or severe, persistent and/or worsening abdominal symptoms, then the standard ICU bowel care will be given and the study drug will be stopped. Stool will be sent to microbiology laboratories for culture and testing for *Clostridium difficile* toxin, if an infective cause is thought clinically likely. The incidence of diarrhoea and *C. difficile* infection is a secondary outcome. Patients will continue in the study, unless consent is withdrawn, and be followed for other end points as part of full analysis and to complete the blood sampling timetable.

Dose modifications for toxicity

▸ In patients with severe renal impairment (estimated glomerular filtration rate (eGFR) <30 mL/min), the dose of methylnaltrexone administered will be reduced to

- 38–61 kg: 4 mg
- 62–114 kg: 8 mg
- Patients who are receiving continuous veno–venous haemofiltration will receive the normal dose.
- The normal dose can be given in mild hepatic impairment, but the study drug is not licensed in severe hepatic impairment (Child Pugh Class C).
- Participants will be followed up daily while on the ICU. Routinely collected clinical data (cardiovascular, respiratory and renal physiological variables as well as haematological, biochemical and microbiological blood test results) will be recorded on a daily basis during this time.
- Patients will also be followed up to ascertain survival status at 28 days postrecruitment and at hospital discharge.

### Outcomes

The primary outcome is time to significant rescue-free laxation following randomisation. Significant laxation is defined as stool volume of >100 mL, as estimated by the attending nurse.

Secondary outcomes include

- Gastric residual volume measured every 4 hours and totalled over 24 hours.
- Toleration of enteral feeds: daily assessment of percentage of patients achieving full target enteral feeding.
- Requirement of rescue laxatives: 1/2 sachet picolax (5 mg sodium picosulfate) and two glycerin suppositories (4 g mould).
- Requirement of prokinetics (10 mg metoclopramide three times a day, 250 mg erythromycin four times a day).
- Average number of bowel movements per day.
- Escalation of opioid dose due to antagonism/reversal of analgesia and sedation.
- Incidence of ventilator-associated pneumonia, defined by the Clinical Pulmonary Infection Score.
- Incidence of diarrhoea.
- Incidence of *C. difficile* infection: PCR or toxin positive.
- Incidence of positive microbiology blood cultures.
- Mortality: 28 days, ICU and hospital.

Exploratory mechanistic outcomes include

- Sepsis biomarkers.
- Leucocyte function tests
- Leucocyte migration assays.

### Participant timeline

See table 1.

**Table 1:** Visit schedule

Visit	Day −1	Day 0	Day 1	Day 2	Day 3	Day 4	Day 5	Days 6–28
Screening	X	X*						
Informed consent†		PerLR/ProLR assent will be obtained initially. This can be done from 24 hours of constipation following admission (though the patient would not be randomised until at least 48 hours have passed). Retrospective patient consent will be obtained when the patient has recovered						
Inclusion/exclusion criteria	X	X*						
Randomisation		X						
Study drug administration			Study drug administered daily until patient has been off opioid sedation for 24 hours or at 28 days					
Blood sampling (15–30 mL)		X	X	X	X	X	X	One further blood sample taken at 24 hours postcessation of opioid infusion
Daily collection of clinical data		X	X	X	X	X	X	X
Final visit			Until patient has been off opioid sedation for 24 hours or at 28 days					

*Main screening for patient if patient has not been screened at day −1 or confirmation of eligibility if patient has been screened at day −1.

†Informed consent will take place if possible between 24 and 48 hours of constipation (at day −1) and if not obtained at day −1 will be obtained at day 0 (48 hours or more of constipation).

Day −1, between 24 and 48 hours of constipation;

Day 0, 48 hours or more of constipation; PerLR, personal legal representative; ProLR, professional legal representative.

### Sample size

The sample size will be 84 patients. The primary end point is time to rescue-free laxation. In a phase III trial in palliative care patients, 48% of participants receiving methylnaltrexone had rescue-free laxation within 4 hours compared to 15% in the placebo arm, p<0.001.^8^ Pilot data in ICU patients suggest that a difference in efficacy of this magnitude would be reasonable in the ICU setting (71% vs 0% opened bowels within 12 hours).^14^ Allowing for a drop-out rate of 5% (patients who withdraw consent after regaining consciousness), with 42 participants in each arm (26 events in total), this study will have 85% power to detect a difference of 33% (15% vs 48%) in the proportion of patients with rescue-free laxation within 12 hours at the 5% level (using a two-tailed log-rank test). This calculation assumes that at the time of analysis, 65% of observations will be censored (either due to withdrawal or rescue), which is likely to be a considerable overestimate since those with rescue-free laxation occurring after 12 hours will also be events. We have nevertheless maintained the sample size at 42 per group, in order to ensure the generalisability of results. The recruitment target will therefore be 84 patients.

### Recruitment

Patients will be reviewed on a daily basis by the unit research nurse. All patients who are clinically constipated and on opioid infusion will be screened against the inclusion and exclusion criteria for eligibility of the study. The initial screening will take place following 24 hours of constipation following admission and opioid infusion. This will then allow for at least another 24 hours to check eligibility criteria and consent from the personal legal representative (PerLR).

## Methods: Assignment of interventions

### Allocation

Randomisation lists (one per ICU) will be prepared using 1:1 allocation (methylnaltrexone vs placebo) by the trial statistician. Appropriate block sizes will be uploaded to InForm (Oracle, California, USA), the study electronic data capture system, prior to the start of the study.

A patient's next of kin will be approached by the recruiting research nurse when the patient is approaching constipation, that is, after 24 hours of constipation while the patient is receiving an opioid infusion and the inclusion and exclusion criteria have been met. The trial outline and information sheet will be given to the patient's next of kin. Provisional written informed consent from the next of kin will be taken for the patient to enter the trial following 48 hours of constipation. Ideally patients will be enrolled immediately after 48 hours, but the enrolment period will remain open following this to account for delays in screening and gaining consent. If consent has not been obtained between 24 and 48 hours of constipation, it will be sought at 48 hours or later and before the patient is randomised into the trial or has any blood samples or data taken for the trial.

Eligible participants will be allocated online to the next available treatment code in the appropriate randomisation list.

### Blinding

When a patient is randomised to the trial, the research nurse will draw up the study drug or placebo into a syringe and the syringe will be labelled to meet the standard hospital requirements before being administered to the patient by the research nurse. The research nurse will remain the only non-blinded member of the team. The bedside nurse, clinical medical team, investigators and the data collection team will be blinded throughout the study.

A randomisation list will be supplied to each hospital pharmacy to allow emergency non-blinding if needed and requested by the local investigators. The local investigators should aim to discuss the need for non-blinding with the trial coordinator or Chief Investigator beforehand if possible, but will have access to a mechanism that permits rapid non-blinding should they feel this is necessary and be unable to contact the study team. Local standard operating procedures (SOPs) describing the emergency non-blinding procedure will be in place. This will be an extremely unlikely situation.

## Methods: Data collection, management and analysis

### Data collection methods

Participants will be followed up daily while in the ICU to ascertain survival status at 28 days postrecruitment and hospital discharge. Routinely collected clinical data (cardiovascular, respiratory, renal and GI physiological variables as well as haematological, biochemical and microbiological blood test results) will be recorded on a daily basis during this time and entered directly by blinded data collection staff onto trial-specific web-based electronic case report forms (eCRFs).

### Data management

Data management will be through the InForm ITM (Integrated Trial Management) System maintained at Imperial Clinical Trials Unit. All personal identifiable data, including those from screened patients, will be kept securely in the local site files and will not be uploaded to the main trial database. InForm generates automatic alerts for missing and invalid data or data that do not conform to the rules established for that data type. There is an electronic audit trail for all data changes. In addition, the central coordinating site will visit local recruiting sites to ensure compliance with the protocol, Good Clinical Practice and local regulatory compliance as well as source data verification.

### Statistical methods

Basic descriptive methods will be used to present the data on study participants, trial conduct, clinical outcomes and safety (in total and for each study group separately). For the primary end point, Cox regression will be used to assess the effect of treatment group on time to rescue-free laxation with ICU included in the model as a random effect to account for stratification. Kaplan-Meier survival curves will also be presented. All efficacy analyses will be on an intention-to-treat basis.

## Methods: Monitoring

### Data monitoring

The Trial Steering Committee (TSC) with an independent Chair, members and two patient and public representatives will be responsible for overseeing the progress of the trial, and will convene six-monthly.

An independent Data Monitoring Committee (DMC) will meet six-monthly to review ongoing recruitment, protocol compliance, safeguard the interests of trial participants, assess the safety and efficacy of the interventions during the trial and monitor the overall conduct of the clinical trial. A separate charter has been drawn up defining their exact remit and criteria for reporting to the TSC. There will be six-monthly meetings of the DMC.

There are no plans for interim analysis. If, in the opinion of the Chief Investigator or DMC, clinical events indicate that it is not justifiable to continue the trial, the TSC may terminate the trial following consultation with the Sponsor.

### Harms

The trial is being conducted on critically ill patients requiring mechanical ventilation. Morbidity and mortality may be expected as a result of their underlying illness. Deaths will, therefore, only be reported as severe adverse events when the investigator deems the event to be related to the administration of the study drug. Details of clinical outcomes will be routinely collected in the eCRF.

All adverse events will be reported. Further guidance will be available from the study coordination centre.

Non-serious adverse reactions such as toxicities, whether expected or not, will be recorded in the toxicity section of the relevant case report form and sent to the study coordination centre within 1 month.

Fatal or life-threatening SAEs and suspected unexpected serious adverse reactions will be reported on the day that the local site is aware of the event. The nature of event, date of onset, severity, corrective therapies given, outcome and causality (ie, unrelated, unlikely, possible, probably, definitely) will be recorded.

An SAE form will be completed and entered in the eCRF for all SAEs within 24 hours of the local site becoming aware of the event. This will automatically send alert emails to the Chief Investigator, the Project Manager and the Sponsor. However, relapse, organ failure and death due to the underlying clinical condition (see definitions above) and hospitalisations for elective treatment of a pre-existing condition do not need reporting as SAEs.

### Auditing

The study may be subject to inspection and audit by Imperial College Academic Health Science Centre under their remit as Sponsor, the Study Coordination Centre and other regulatory bodies to ensure adherence to Good Clinical Practice (GCP).

## Ethics and dissemination

### Research ethics approval

The trial protocol, the patient and PerLR information sheets, and consent forms have been reviewed and approved by the Harrow Research Ethics Committee (REC Reference 14/LO/2004). Clinical Trial Authorisation from the Medicines and Healthcare Products Regulatory Agency has been obtained.

### Protocol amendments

Proposed amendments to the protocol and aforementioned documents will be submitted to the REC for approval as instructed by the Sponsor. Amendments requiring REC approval may be implemented only after a copy of the REC's approval letter has been obtained. Amendments that are intended to eliminate an apparent immediate hazard to participants may be implemented prior to receiving Sponsor or REC approval. However, in this case, approval must be obtained as soon as possible after implementation. The regulatory authorities and REC will be sent annual progress reports and informed about the end of trial, within the required timelines.

### Consent

As patients will be sedated with opioids to facilitate mechanical ventilation, it will not be possible to obtain prospective consent from the patient at the time of enrolment. As all the study drugs are already routinely used in the management of constipation, there is minimal extra risk from participation in this study.

### PerLR consent

As the patient is unable to give consent, informed consent will be sought from the patient's PerLR who may be a relative, partner or close friend. The PerLR will be informed about the trial by the responsible clinician or a member of the research team and provided with a copy of the PerLR Information Sheet and asked to give an opinion as to whether the patient would object to taking part in such medical research. The PerLR will be approached following 24 hours of OIC and will be given a further period of time to consider the patient's participation in the study. If the PerLR decides that the patient would have no objection to participating in the trial, they will be asked to sign the PerLR Consent Form that will then be counter signed by the responsible member of the research team. The PerLR will retain a copy of the signed consent form. The patient, if still suffering from OIC, will then be suitable for entry into the trial at 48 hours of OIC. Patients who laxate between 24 and 48 hours will not be entered into the trial, but routine data collected as part of their intensive care stay may be compared to the study group.

### Professional legal representative consent

If the patient is unable to give informed consent, and attempts to meet and discuss with a PerLR have failed, then a doctor who is not connected with the conduct of the trial may act as a professional legal representative (ProLR). The doctor will be informed about the trial by a member of the research team and given a copy of the ProLR Covering Statement. If the doctor decides that the patient is suitable for entry into the trial, they will then be asked to sign the ProLR consent form. Subsequently, if a relative, partner or close friend visits the patient before he or she has regained consciousness, then they should be informed about the patient's participation and also informed about the retrospective consent process.

### Retrospective patient information

If and when the patient recovers and they regain the capacity to understand the details of the trial, a member of the research team will inform them of their participation in the trial. The patient will be given a copy of the Patient Information Sheet. The patient will be asked for consent to continue participation in the trial and to sign the Retrospective Consent Form. If the patient does not want to continue participation in the study, they will be given the choice of having the already collected data and samples excluded from the final analysis.

The right of the participant or their PerLR to refuse to participate without giving reasons must be respected. After the participant has entered the trial, the clinician remains free to give alternative treatment to that specified in the protocol at any stage if he/she feels it is in the participant's best interest, but the reasons for doing so should be recorded. In these cases, the participants remain within the study for the purposes of follow-up and data analysis. All participants are free to withdraw at any time from the protocol treatment without giving reasons and without prejudicing further treatment.

### Confidentiality

Participants' identification data (initials and date of birth) will be required for the registration process. The Study Coordination Centre will preserve the confidentiality of participants taking part in the study and is registered under the Data Protection Act.

The investigator will ensure that the participants' privacy is maintained. On the eCRF or other documents submitted to the Sponsor, participants will be identified by a participant ID number only. Documents that are not submitted to the Sponsor (eg, signed informed consent forms) will be kept in a strictly confidential file by the investigator.

The investigator shall permit direct access to participants' records and source documents for the purposes of monitoring, auditing or inspection by the Sponsor, authorised representatives of the Sponsor, regulatory authorities and RECs.

### Access to data

The investigator will retain essential documents until notified by the Sponsor, and at least for 10 years after study completion, as per the Sponsor's SOPs. Participant files and other source data (including copies of protocols, CRFs, original reports of test results, correspondence, records of informed consent and other documents pertaining to the conduct of the study) will be kept for the maximum period of time permitted by the institution. Documents will be stored in such a way that they can be accessed/data retrieved at a later date. Consideration will be given to security and environmental risks.

No study document will be destroyed without prior written agreement between the Sponsor and the investigator. Should the investigator wish to assign the study records to another party or move them to another location, written agreement will be obtained from the Sponsor.

Source documents include original documents related to the trial, to medical treatment and to the history of participants, and will be maintained to allow reliable verification and validation of the trial data.

### Disseminated policy

All publications and presentations relating to the study will be authorised by the Trial Management Group. Authorship will be determined according to the internationally agreed criteria for authorship (http://www.icmje.org). Authorship of parallel studies initiated outside of the Trial Management Group will be according to the individuals involved in the project but must acknowledge the contribution of the Trial Management Group and the Study Coordination Centre.

Data and all appropriate documentation will be stored for a minimum of 10 years after the completion of the study, including the follow-up period.
